# EANM guideline for ventilation/perfusion single-photon emission computed tomography (SPECT) for diagnosis of pulmonary embolism and beyond

**DOI:** 10.1007/s00259-019-04450-0

**Published:** 2019-08-13

**Authors:** Marika Bajc, Carl Schümichen, Thomas Grüning, Ari Lindqvist, Pierre-Yves Le Roux, Adriano Alatri, Ralf W. Bauer, Mirza Dilic, Brian Neilly, Hein J. Verberne, Roberto C. Delgado Bolton, Bjorn Jonson

**Affiliations:** 1 Department of Clinical Sciences, Clinical Physiology and Nuclear Medicine, University of Lund, Lund, Sweden; 2 University of Rostock, Formerly Clinic for Nuclear Medicine, Rostock, Germany; 3 Department of Nuclear Medicine, University Hospitals Plymouth, Plymouth, UK; 4 Research Unit of Pulmonary Diseases, Clinical Research Institute, HUS Helsinki University Hospital, Helsinki, Finland; 5 Department of Nuclear Medicine, University Hospital of Brest, Brest, France; 6 Division of Angiology, Heart and Vessel Department, Lausanne University Hospital, Lausanne, Switzerland; 7 RNS Gemeinschaftspraxis, Wiesbaden, Germany; 8 Department of Diagnostic and Interventional Radiology, Goethe University Frankfurt (Main), Frankfurt, Germany; 9 Clinic of Heart and Blood Vessel Disease, Clinical Center University of Sarajevo, Sarajevo, Bosnia and Herzegovina; 10 Department of Nuclear Medicine, Royal Infirmary, Glasgow, UK; 11 Department of Radiology and Nuclear Medicine, Amsterdam UMC, Location AMC, University of Amsterdam, Amsterdam, The Netherlands; 12 Department of Diagnostic Imaging (Radiology) and Nuclear Medicine, University Hospital San Pedro and Centre for Biomedical Research of La Rioja (CIBIR), Logroño, La Rioja Spain

**Keywords:** Pulmonary embolism, Ventilation-perfusion, SPECT, CTPA, V/P SPECT/CT, COPD, Left heart failure, Pneumonia, Chronic pulmonary embolism, Pulmonary hypertension

## Abstract

These guidelines update the previous EANM 2009 guidelines on the diagnosis of pulmonary embolism (PE). Relevant new aspects are related to (a) quantification of PE and other ventilation/perfusion defects; (b) follow-up of patients with PE; (c) chronic PE; and (d) description of additional pulmonary physiological changes leading to diagnoses of left ventricular heart failure (HF), chronic obstructive pulmonary disease (COPD) and pneumonia. The diagnosis of PE should be reported when a mismatch of one segment or two subsegments is found. For ventilation, Technegas or krypton gas is preferred over diethylene triamine pentaacetic acid (DTPA) in patients with COPD. Tomographic imaging with V/P_SPECT_ has higher sensitivity and specificity for PE compared with planar imaging. Absence of contraindications makes V/P_SPECT_ an essential method for the diagnosis of PE. When V/P_SPECT_ is combined with a low-dose CT, the specificity of the test can be further improved, especially in patients with other lung diseases. Pitfalls in V/P_SPECT_ interpretation are discussed. In conclusion, V/P_SPECT_ is strongly recommended as it accurately establishes the diagnosis of PE even in the presence of diseases like COPD, HF and pneumonia and has no contraindications.

## Preamble

The European Association of Nuclear Medicine (EANM) will periodically develop new guidelines for nuclear medicine practice to promote the science of nuclear medicine and to improve the quality of service to patients throughout Europe. Each practice guideline, representing an EANM policy statement, has undergone an extensive consensus and review process.

The EANM has written and approved these guidelines to promote the use of nuclear medicine procedures of high quality. These guidelines are intended to assist practitioners in providing appropriate nuclear medicine care for patients. They are not inflexible rules or requirements of practice and are not intended, nor should they be used, to establish a legal standard of care.

The ultimate judgment regarding the propriety of any specific procedure or course of action must be made by medical professionals considering the unique circumstances of each case. Thus, there is no implication that an approach differing from the guidelines is below the standard of care. On the contrary, a conscientious practitioner may responsibly adopt a course of action different from that set forth in the guidelines when, in the reasonable judgment of the practitioner, such course of action is indicated by the condition of the patient, limitations of available resources or advances in knowledge or technology subsequent to publication of the guidelines.

## Introduction

These guidelines update the 2009 EANM guidelines on the diagnosis of pulmonary embolism (PE) [1, 2] for ventilation/perfusion single-photon emission tomography (V/P_SPECT_). Since the previous EANM guidelines, little new data has emerged with regard to the technical aspects of V/P_SPECT_. This document defines the role of V/P_SPECT_ in the diagnosis of PE and other cardiopulmonary diseases.

## Pulmonary embolism

Nonthrombotic emboli may be septic, fat, amniotic fluid and air. In this document, PE refers to venous thromboembolism (VTE). PE is an important and treatable illness caused by migration of thrombi to the pulmonary circulation, commonly from the veins of the lower extremities (deep vein thrombosis: DVT). PE can cause death in the acute phase or later through chronic thromboembolic pulmonary hypertension.

Although independent VTE risk factors and predictors of VTE recurrence have been identified, and effective primary and secondary prophylaxis is available, the occurrence of VTE seems to be relatively constant, or even increasing [3]. Timely and accurate PE diagnosis is, therefore, essential.

### Natural history of PE

The natural history of VTE has been extensively studied. Measurements of fibrinogen uptake were used by Kakkar et al. in the study evidencing that DVT developed in 30% of 132 patients undergoing surgery without prophylaxis [4]. In most patients, DVT developed in the calf veins, propagating to the proximal leg veins in 13%. Forty-four percent of patients with proximal DVT developed PE. Evidence that DVT and PE are distinct manifestations of the same disease process, referred to as VTE, has been provided by observations showing that in the majority of patients with PE, DVT can be detected using sensitive methods. In patients with proven leg vein DVT, 40% have asymptomatic PE [5]. However, whereas VTE can present with one or both of these two manifestations, DVT and PE, epidemiological differences between both are important. Mortality is higher for PE than for DVT [6]. The International Cooperative Embolism Registry [7] aimed at determining baseline mortality rates and mechanisms of death reported a 3-month overall mortality rate of 15%; the factors significantly associated with increased mortality being systolic arterial hypotension, congestive heart failure, cancer, tachypnoea, right ventricular hypokinesia, COPD and age > 70 years. Resolution of PE is variable. Evidence shows that a majority of patients have unresolved PE at 6 months from diagnosis [8]. Others report rapid resolution of a large PE within hours of the onset of heparin therapy [9]. Fredin and Arborelius evidenced complete restoration of lung perfusion in patients with PE within 1 week of diagnosis [10]. Based on this rapidly changing pattern of perfusion in PE, Coakley recommended that imaging tests for PE diagnosis should be carried out as soon as possible, preferably within 24 h after onset of symptoms [11].

### Epidemiology

Venous thromboembolism is a major cause of morbidity, mortality and hospitalisation [3, 12–14]. A model based on data from 6 European countries with a combined population of 310 million found approximately 466,000 cases of DVT and 296,000 cases of PE in 2004. These resulted in approximately 370,000 deaths, of which 7% were thought to be from diagnosed and treated VTE, 34% from sudden fatal PE and 59% from PE following undiagnosed VTE [3]. Incidence for DVT and PE increases with age. VTE is rare prior to late adolescence [12, 15, 16].

One-third of PE episodes occur without any known risk factor and are classified as ‘unprovoked’ [17]. The remaining ‘provoked’ PE episodes are secondary to a risk factor that may be temporary (e.g. surgery, trauma, immobilisation, pregnancy, oral contraceptive or hormone replacement therapy) or persistent (e.g. cancer or inherited thrombophilia) [12, 18, 19]. About 20% of all VTEs are cancer-related [20]. Surgery and immobilisation each account for 15% of cases [21]. Not infrequently, PE is without clinical manifestations [22].

The most frequent inherited risk factors are factor V and prothrombin (factor II) gene mutations. These have a European prevalence of 3–7% and 1–2%, respectively [23].

Following a PE, about one-third of patients show persistent pulmonary perfusion defects [24–26]. Chronic thromboembolic pulmonary hypertension (CTEPH) is the principal long-term complication of PE with an incidence of 0.1–4% [27].

PE frequently recurs, usually after discontinuation of anticoagulation (13% at 1 year, 23% at 5 years and 30% at 10 years) [12]. Recurrence rate is higher after unprovoked VTE than after provoked VTE and higher after multiple episodes compared with a single event [12].

### Pathophysiology

When unperfused regions are ventilated, there is an increase in the dead space [28]. This is one of the reasons for dyspnoea. Hypoxia, frequently present in major PE, is caused by several mechanisms. The emboli occluding pulmonary end arteries alter the local equilibrium and, therefore, can lead to haemorrhage, atelectasis, pleural effusion and pleuritic pain. The lung has no pain fibres; thus, pain in PE is a symptom consequence of the involvement of the parietal pleura.

Moreover, there is also increased pulmonary vascular resistance that can produce right ventricular strain and failure, electromechanical dissociation, hypotension, syncope and sudden death. Pressure increase in the right atrium may lead to right-to-left shunt through a patent foramen ovale, contributing to hypoxaemia. The shunt can also produce paradoxical emboli, resulting in infarctions from venous thrombi in the systemic circulation, commonly the brain [29–31].

### Clinical presentation

The clinical signs of PE range from asymptomatic to sudden death [12, 22]. Most patients with PE have symptoms including dyspnoea, tachypnoea, chest pain (pleuritic or retrosternal), cough, fever, haemoptysis, syncope, unilateral leg pain or swelling, palpitations, tachycardia or dizziness due to hypotension [32, 33]. Arterial hypotension and shock are rare signs indicating central massive PE and/or a severely reduced haemodynamic reserve, and these clinical signs and symptoms indicate high-risk PE. It is associated with particularly high early mortality [31]. In the case of central PE, chest pain may have the characteristics of angina, probably because of right ventricular ischemia, and poses the problem of differentiating PE from acute coronary syndrome and aortic dissection [31].

The clinical features of PE are also common in patients without PE [32, 33], and the prevalence of PE in patients with clinically suspected VTE is only about 20% [34, 35]. Therefore, there is a risk that an undue number of patients might receive an unnecessary imaging procedure. Thus, assessments of clinical PE probability and d-dimer testing are important steps in clinical practice to guide decisions about who should be referred for imaging [31, 36]. The chest radiograph is useful for alternative diagnoses such as pneumothorax, pneumonia, COPD, lung cancer or pulmonary fibrosis.

### Assessment of pretest clinical probability

Clinical probability for PE can be assessed empirically by clinical judgement (holistically) or by clinical prediction rules, foremost Wells’ and the revised Geneva scores [37, 38], which have been adequately validated [34, 35, 39] and recommended by EANM, the Society of Nuclear Medicine and Molecular Imaging and the European Society of Cardiology (ESC) [31, 40]. For both scores, simplified and validated versions are available [39, 41–44]. Usually, patients are clinically stratified into two or three risk categories: unlikely/likely and low/intermediate/high with increasing prevalence of PE [45].

### d-dimer testing

Plasma d-dimer (a breakdown product of fibrin clot) is not only regularly elevated in patients with venous thromboembolism but also in myocardial infarction, stroke, infection, inflammation, cancer and pregnancy. The specificity of d-dimer is, therefore, low, and a positive d-dimer test does not confirm PE. However, the d-dimer test is very sensitive. Therefore, when the d-dimer is below a predefined cut-off value (i.e. < 500 μg/L with correction for age), it can be used to exclude PE in patients with either low to intermediate or unlikely clinical probability [31, 46]. However, in old and persistent PE, d-dimer can be negative. In patients with a high or likely clinical probability, the d-dimer has no discriminating power and should not be measured [47].

In a recent randomised clinical trial including 1916 patients with suspected PE, considered by clinical judgement (holistically) to be at very low risk for PE, the use of the pulmonary embolism rule-out criteria (PERC) safely excluded PE [48]. The PERC strategy may reduce the number of d-dimer tests in patients with very low clinical probability of PE [48], although caution is advised in using the PERC rule [49].

### Imaging tests

In patients with a low or intermediate clinical probability but positive d-dimer, and in patients with a high or likely clinical probability, lung imaging is required. The two mainly used imaging modalities are as follows:

- V/P imaging with SPECT (V/P_SPECT_) or in rare situations planar scintigraphy (V/P_planar_). Occasionally, perfusion-only lung scanning is performed. V/P_SPECT_ may also be combined with low-dose computed tomography (CT), V/P_SPECT/CT_
- Computed tomography of the pulmonary arteries (CTPA)

Invasive pulmonary angiography is no longer regarded as the gold standard for the diagnosis of PE because of its limited sensitivity and specificity and wide interobserver variability [50].

Transthoracic echocardiography was discussed in the former guidelines in haemodynamically unstable patients [1, 2]. In a recent review, the need for further research on this topic is underlined [51].

### Basic principles of PE diagnosis

**Fig. 1:**
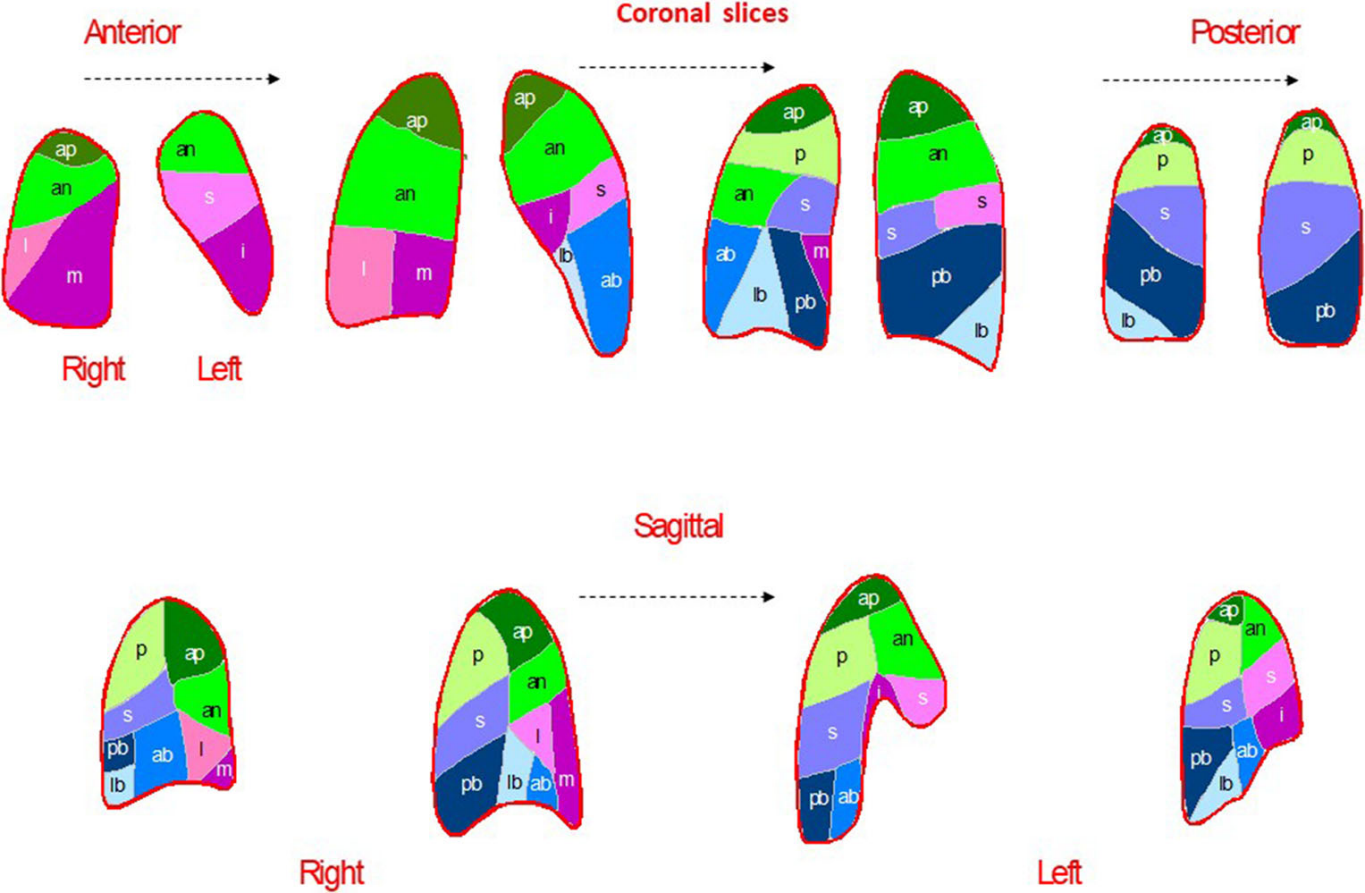
Segmental map of the two lungs in four coronal slices and two sagittal slices for each lung. A bronchopulmonary segment is conical with its apex towards the hilum and its base projected onto the pleural surface. Thrombi occluding pulmonary arteries, therefore, produce characteristic lobar, segmental or subsegmental peripheral wedge-shaped defects with the base reaching the pleural surface

**Fig. 2:**
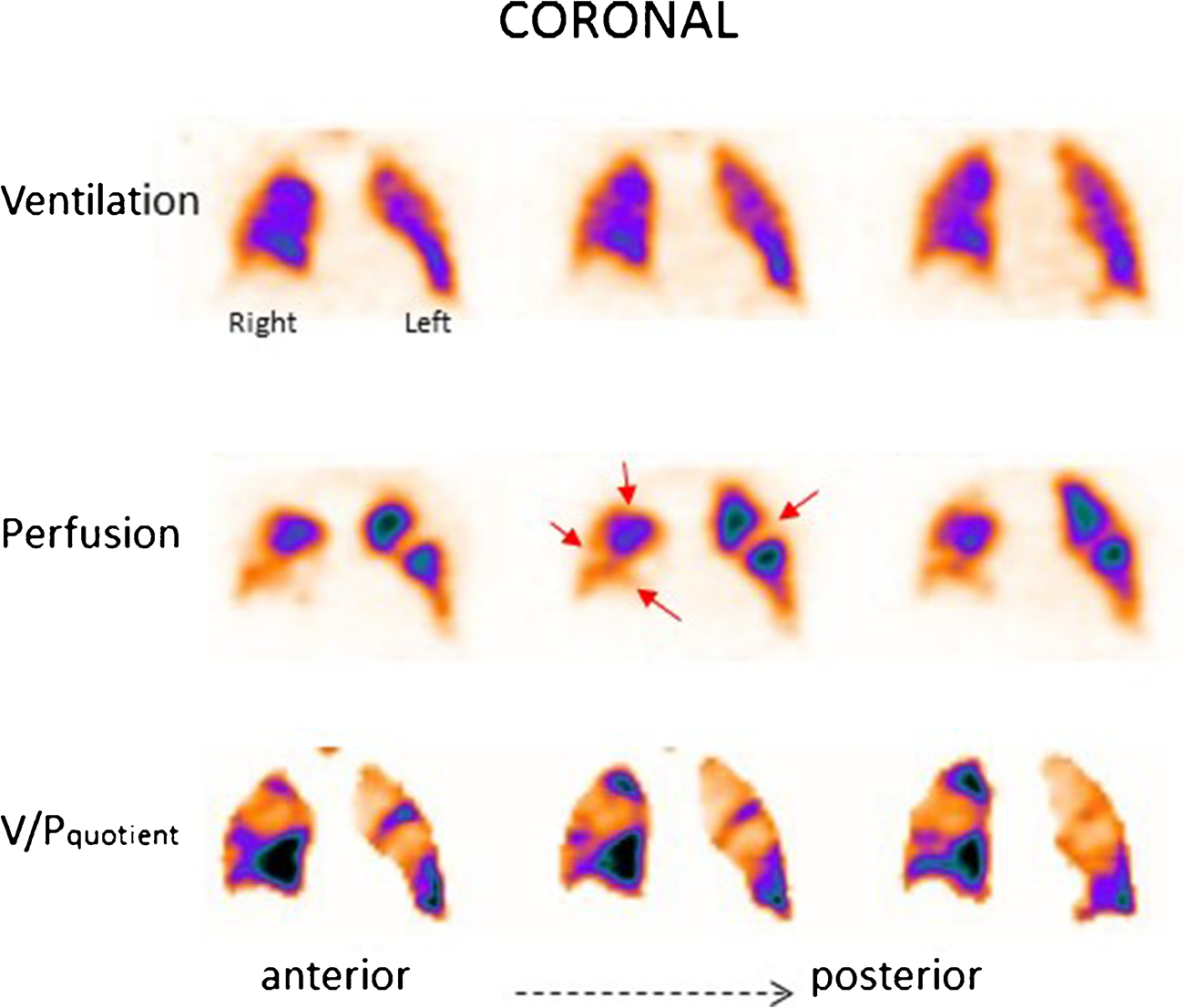
Coronal slices in a patient with PE. Multiple bilateral segmental perfusion defects (red arrows) in areas with normal ventilation. These are delineated on V/P quotient images which facilitate interpretation

PE leads to loss of perfusion to the area corresponding to the volume supplied by the occluded end artery that may be a whole lung, a lobe, a lung segment or a subsegment. In general, the bronchial circulation maintains viability of the embolised volume, and ventilation remains largely intact. Accordingly, V/P_SPECT_ exploits the unique pulmonary arterial segmental anatomy. Figure 1 presents a segmental map, and a case with PE is shown in Fig. 2.

PE is in general an acute disease that should be diagnosed and treated without delay. V/P_SPECT_ should, therefore, be performed according to a 1-day protocol. V/P_planar_ should only be used when a patient for any reason cannot be examined by V/P_SPECT_.

## Radiopharmaceuticals for V/P_SPECT_

### Ventilation

Ventilation can be evaluated with the ^99m^Tc-labelled aerosols, DTPA and Technegas®, or krypton gas (^81m^Kr) (Table 1).

**Table 1:** Dosimetry of radiopharmaceuticals used for V/P_SPECT_

Radiopharmaceutical	Administered activity (MBq)	Critical organ (mGy/MBq)	Effective dose (mSv/MBq)
^99m^Tc-MAA [52]	40–120	0.067 lungs	0.017
^99m^Tc-DTPA [53]	20–30	0.047 bladder	0.007
^99m^Tc-Technegas [54]	20–30	0.11 lungs	0.015
^81m^Kr [55]	40–400	0.0068 lungs	0.0007

The biological half-life of ^99m^Tc-DTPA is 55–108 min [56] and of ^99m^Tc-Technegas 135 h [57]

^99m^Tc-DTPA (^99m^Tc-diethylen-tetraamino-pentaacetate) is aerosolised from a water solution with a particle size of 1.2–2 μm. ^99m^Tc-DTPA allows studies of alveolo-capillary permeability [58].

Technegas® is an aerosol of ^99m^Tc-labelled solid graphite hydrophobic particles, with a diameter of about 0.005–0.2 μm [59]. The particles tend to grow by aggregation and should, therefore, be used within 10 min after generation [60]. The particle size is so small that the aerosol behaves nearly like a gas until it arrives at the periphery of the lung where the particles are deposited in bronchioli and alveoli, mostly by diffusion. Technegas® greatly reduces the problem of central deposition often encountered with ^99m^Tc-DTPA. Technegas® facilitates interpretation, particularly in COPD [58]. Hotspots are nevertheless seen in patients with severe airway obstruction. The penetration index for Technegas® may be used for grading of COPD severity [61, 62].

Krypton gas (^81m^Kr) is an inert radioactive gas delivered from an ^81^Rb/^81m^Kr generator. ^81m^Kr has a half-life of 13 s. ^81m^Kr is inhaled until it reaches a steady-state activity in the alveoli and then continuously during the whole imaging procedure. Due to the higher gamma energy (190 keV) of ^81m^Kr compared with ^99m^Tc (140 keV), ventilation and perfusion images can be acquired simultaneously. Because elimination of ^81m^Kr from the alveoli is largely due to decay of the isotope rather than by expiration, regional activity at steady state accurately represents regional ventilation. In COPD, the inhalation time to reach steady state may, however, be too long to reach steady state. As the half-life of ^81^Rb is only 4.6 h, the need for daily delivery of the expensive cyclotron-produced generator limits the clinical use of ^81m^Kr.

### Perfusion

For perfusion scintigraphy, intravenously injected macroaggregates of ^99m^Tc-labelled human albumin (MAA) with a diameter of 15–100 μm are nearly universally used. Intravenous injection of MAA leads to microembolisation of pulmonary precapillary arterioles and capillaries. Whilst 60,000 particles may suffice to reflect regional perfusion [63], about 400,000 labelled particles are usually injected. This leads to obstruction of a very small fraction of pulmonary vessels. Injection of no more than 100,000–200,000 particles is recommended for patients with known pulmonary hypertension, right-to-left heart shunt, pneumonectomy or after single lung transplantation. In infants and children, the number of particles is recommended to be further reduced according to weight [64].

Because of religious beliefs, it may be advisable to inform patients that MAA is a blood product.

### Quality control and injection practice

As radiochemical purity varies, supernatant activity should be determined. As particles tend to settle, the vial should be shaken before use. Withdrawal of blood into the syringe should be avoided, as this will cause aggregation of MAA particles resulting in artefactual hotspots. The MAA suspension should be injected over 30 s, i.e. over several breaths, to promote distribution reflecting regional pulmonary perfusion. Patients should be in a supine position during inhalation, intravenous injection and during scanning.

## Imaging protocols

Using aerosols, imaging starts with the ventilation scan, immediately followed by the perfusion scan, according to principles based on an extensive analysis serving to optimise radioactivity doses, the relationship between activities and scanning time for ventilation and perfusion, type of collimator, number of rotational steps, matrix size and image reconstruction algorithm [65] (Table 2). Activities of 25–30 MBq and 140–160 MBq are sufficient for ventilation and perfusion studies, respectively, with a general-purpose collimator, 64 × 64 matrix and 60–64 rotational steps of a dual head gamma-camera, i.e. 120–128 projections [65, 66]. Time per projection should be 10 s for ventilation and 5 s for perfusion studies.

**Table 2:** Summary of ventilation/perfusion protocol for V/P_SPECT_. Patients should be in a supine position during inhalation, intravenous injection and during scanning

	Ventilation	Perfusion
Administration	Inhalation	Intravenous injection
Radiopharmaceutical administered activity	Technegas® or DTPA 25–30 MBq to reach the lung	^99m^Tc-MAA 120–160 MBq
Particle size	0.005–0.2 μm or 1.2–2 μm	15–100 μm
Time of imaging	≈ 11 min	≈ 5 min
Acquisition protocol	General purpose collimator: 64 × 64 matrix, 60–64 steps for each head, 5 s/step (V) 10 s/step (P) [65, 66]	
Reconstruction	Iterative reconstruction, e.g. OSEM with 8 subsets and 4 iterations	

This principle leads to the lowest radiation exposure consistent with adequate image quality and, therefore, is in accordance with good medical practice. It is applied in commercially available software and hardware systems and is recommended in these guidelines. Deviations from these validated standards require complementary documentation. The use of low-energy high-resolution (LEHR) collimators would require either longer acquisition times or higher activities.

Iterative reconstruction is essential, e.g. ordered subset expectation maximisation (OSEM). For comparison between ventilation and perfusion including triangulation, the patient must be in the same position for the whole image acquisition.

Perfusion-only scintigraphy is recommended as a first step during the first 3 months of pregnancy and in the case of suspected massive PE (see below).

### Image presentation

Standard software allows image presentation in coronal, sagittal and transverse projections as well as the presentation of rotating 3D images (Segami, Hermes, GE and others). Ventilation/perfusion quotient images may be derived from the primary images [65, 66]. Ventilation counts are normalised to perfusion counts before V/P quotient images are calculated. V/P quotient images facilitate diagnosis and quantification of PE extension. As attenuation is the same for ventilation and perfusion, V/P images do not require attenuation correction.

For quality control and fast orientation, an overview of ventilation and perfusion in coronal and sagittal slices is useful. For identification of matched and mismatched ventilation and perfusion changes and particularly for calculation of V/P quotient images, it is essential that ventilation and perfusion images are carefully aligned to each other. An example is shown in Fig. 2 of a patient with PE and multiple segmental perfusion defects, well delineated on V/P quotient images. This is facilitated by the one-session protocol with the patient in an unchanged position. Although helpful, V/P quotient images are not a prerequisite for high quality V/P_SPECT_. The option to triangulate between coronal, sagittal and transverse slices is valuable.

## Interpretation and reporting of findings

### Ventilation/perfusion patterns

For V/P_SPECT_, interpretation criteria are as important as the imaging technique itself. Studies based on probabilistic reporting of planar imaging (PIOPED I) show high rates of nondiagnostic reports. In contrast, V/P_SPECT_ with holistic interpretation (Table 3) is associated with very low rates of nondiagnostic reports and allows a diagnostic conclusion that is binary with respect to PE. All ventilation and perfusion patterns as well as the extent of defects should be described. A few patients might show widespread V/P abnormalities not specific for any disease.

**Table 3:** Semiology of lung ventilation/perfusion pathology with V/P_SPECT_. Interpretation and differential diagnosis

Semiology			Diagnosis	Probability that the semiologic pattern is diagnostic	Level of evidence
Pattern	Distribution	Area			
Mismatch	Segmental	1 segment	PE*	Very high	
		≥ 2 subsegments	PE*	Very high	
		≤ 1 subsegment	Non-PE	High	
	Total lung unperfused	Total lung	Tumour^#^	High	
			Abscess^#^	High	
			Massive PE	Very low (rare)	Very rare condition
	Nonsegmental	Systematically antigravitational redistribution	Heart failure	High	
		Irregular	Vasculitis	Very low (rare)	Very rare condition, expert opinion
Match	Segments or lobules	No stripe sign	Tumour	Depends on clinical context	
			Atelectasia		
			Empyema		
		Stripe sign	Pneumonia evolutioned stage	High	
Reverse mismatch^§^	Segments or lobules	Stripe sign	Pneumonia initial stage	High	
		No stripe sign	COPD	High	

*Even in the presence of concomitant pathologies

^#^Recommend CTPA

^§^More reduction of ventilation than perfusion

### Criteria for acute pulmonary embolism

The recommended basic criteria for reading V/P scintigraphy are the following:

**Table:** 

PE: • V/P mismatch of at least one segment or two subsegments in keeping with the pulmonary vascular anatomy (wedge-shaped defects with the base projecting to the lung periphery).	
No PE: • Normal perfusion pattern in keeping with the anatomic boundaries of the lungs. • Matched or reversed-mismatched V/P defects of any size, shape or number in the absence of mismatch. • Mismatch that does not follow a lobar, segmental or subsegmental pattern.	
Nondiagnostic for PE: • Widespread V/P abnormalities not typical of specific diseases.	

As proposed in the 2009 EANM guidelines, the diagnostic cut-off to consider a V/P_SPECT_ positive for PE should be 1 segmental or 2 subsegmental mismatched defects. This principle is further supported [67], and its safety was confirmed in large studies [67–69]. A single subsegmental mismatched perfusion defect should be reported but does not fulfil diagnostic criteria for PE. In some cases, PE may be found on CTPA in such patients, but clinical significance is not documented. Pulmonary arteries and capillary beds uniquely possess fibrinolytic properties that both trap and lyse small subsegmental clots, suggesting that small PEs are a common physiological phenomenon.

Recommendations from the ESC suggest an individualised approach; patients can be managed conservatively if the presence of deep vein thrombosis has been excluded [56]. Patients with a Wells score > 4 have a 4-fold increased risk of adverse outcome with one or multiple emboli in subsegmental arteries [70].

Applying these principles of interpretation, recent V/P_SPECT_ studies amounting to over 5000 cases report a negative predictive value of 97–99%, sensitivities of 96–99% and specificities of 96–98% for PE diagnosis. Rates of nondiagnostic findings were 1–4% [61, 68, 69, 71–78].

### Pitfalls in the interpretation of V/P_SPECT_

As with any diagnostic test, it is vital that the reporting physician has the knowledge of a number of sources of error. These include the following:

- Technical artefacts may arise from preinjection handling of the ^99m^Tc-MAA. The withdrawal of blood into the syringe that contains the solution of ^99m^Tc-MAA can cause the aggregation of particles that can produce hotspots in the images. A similar consequence can appear from failure to resuspend ^99m^Tc-MAA particles before the administration.
- Planar imaging may underestimate the presence or extent of perfusion abnormalities because of normal perfusion masking embolised regions, also known as the shine through of normal areas. This problem is eliminated by V/P_SPECT_.
- Technegas® is preferred over liquid aerosols in patients with COPD. Moreover, in rare patients with emphysema, Technegas® particles are trapped in bullae in which perfusion is absent. This causes a pattern that may be mistaken for a mismatch [76, 79].
- In rare cases, vasculitis and congenital vascular anomalies may lead to segmental/lobar mismatches.
- Mismatched perfusion defects without a clear segmental character may be seen in older, partly resolved PE, but not related to acute PE. These nonsegmental mismatched defects are observed in several lung disorders including lung cancer, mediastinal lymphadenopathy, postradiation pneumonitis/fibrosis and heart failure. V/P_SPECT_ facilitates the identification of segmental perfusion defects, which are particularly well visualised when using rotating 3D volumetric images.
- Unilateral absence of perfusion in a whole lung with preserved ventilation and without any V/P mismatch in the other lung is generally not due to PE [80, 81]. In such cases, chest CT may reveal the presence of other pathologies such as tumour, aortic dissection, other mediastinal processes or congenital pulmonary vascular abnormalities.
- The ‘rind’ artefact seen in ventilation SPECT represents a band of increased activity along the posterior (dependent) portion of the lung. This is probably produced by dynamic changes in the lung volume during the acquisition [57, 82].
- Fissure artefacts are a common finding along the line of the oblique fissure, especially in perfusion SPECT. It may produce a nonsegmental mismatch [57].

## Additional considerations

### Quantification of PE extent

The extent of PE is an independent risk factor for PE recurrence [83–85]. Quantification of PE may be useful for the management of patients with acute PE [86]. V/P_SPECT_ is particularly suitable for quantification. The number of segments and subsegments indicating mismatch typical of PE can be counted and expressed as a percentage of the total lung parenchyma. However, as the volume of each segment and subsegment is different, this calculation is approximate providing a semiquantification of the percentage of the total lung parenchyma affected. Furthermore, areas with ventilation abnormalities can be recognised allowing for the assessment of the degree of total lung function affected. In haemodynamically stable patients with PE, outpatient management is safe provided that the embolic burden, quantified using V/P_SPECT_, is included in the treatment decision algorithm [86].

### Follow-up

**Fig. 3:**
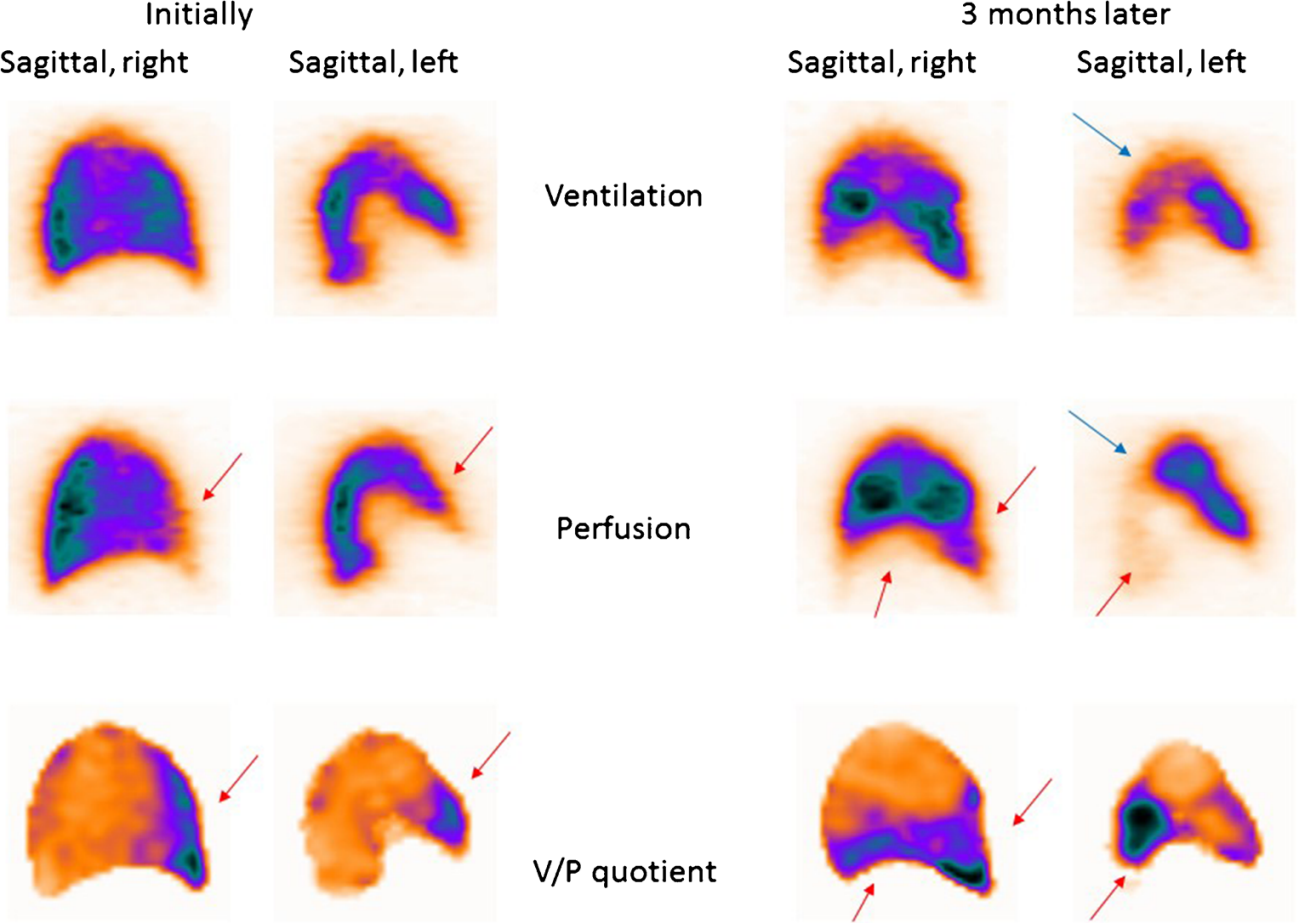
Sagittal slices of both lungs in a patient with small PE (red arrows), initially (not treated) and 3 months later. On follow-up, the progression of perfusion defects as well as deterioration in the ventilation (blue arrow in the left lung) can be seen clearly. The patient also developed pneumonia

The natural history of PE is still not well known. Alternative strategies for PE therapy should be studied, regarding therapy duration and choice of drugs in different categories of patients. The issue of small emboli is particularly relevant, as the need for treatment is not properly supported by evidence. Figure 3 presents a patient with untreated small PE who developed chronic PE after 3 months of follow-up.

Follow-up of PE using imaging is essential to:

- Assess therapy effect
- Differentiate between new and old PE when there is a suspicion of PE recurrence
- Explain physical incapacity after PE

**Fig. 4:**
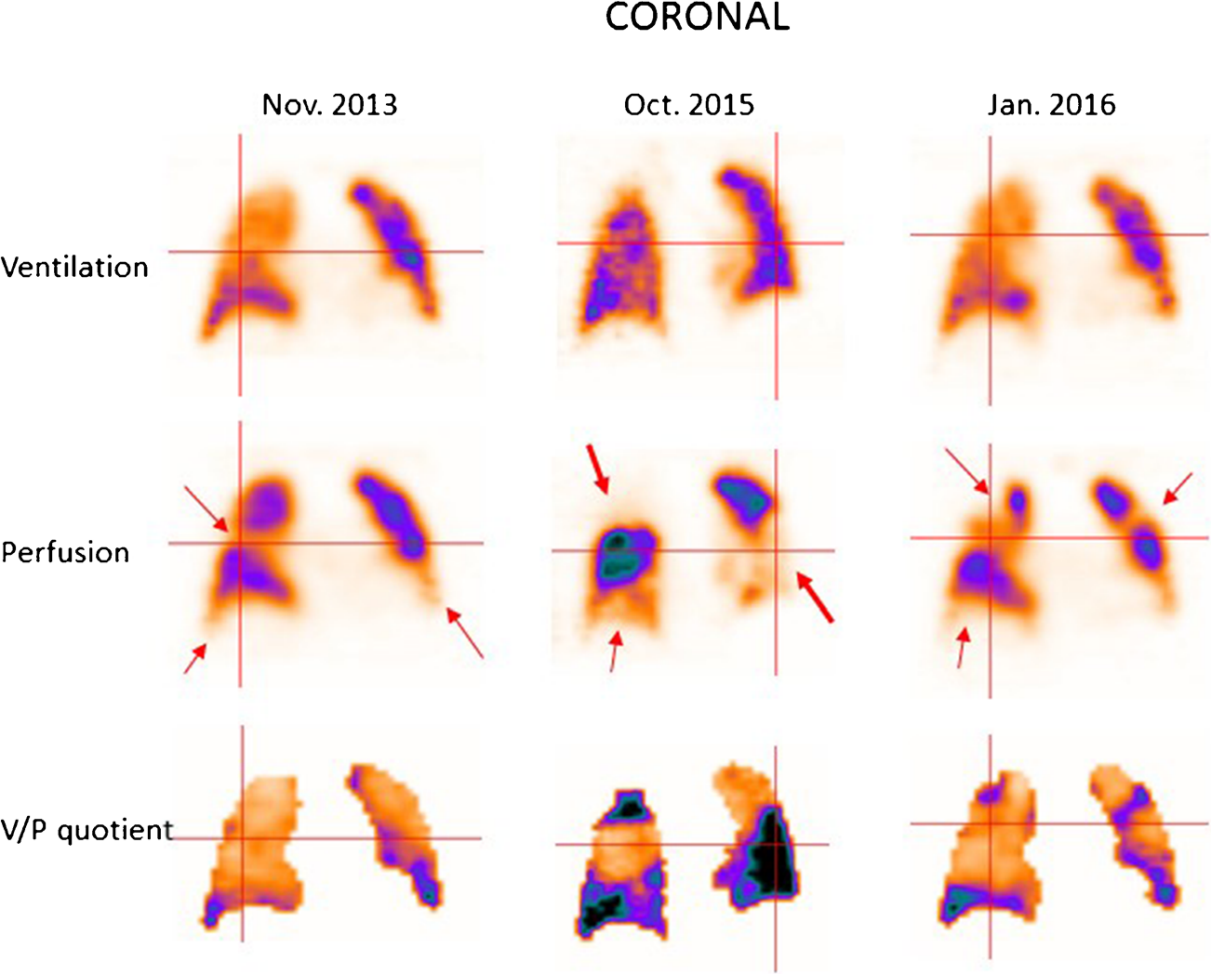
Coronal slices in a patient with recurrent PE (red arrows), initially referred after a diagnosis of pulmonary hypertension. PE was not identified on CT. The first follow-up scan shows new perfusion defects that occurred after stopping therapy, and the final follow-up identifies improving perfusion after 4 months of treatment

**Fig. 5:**
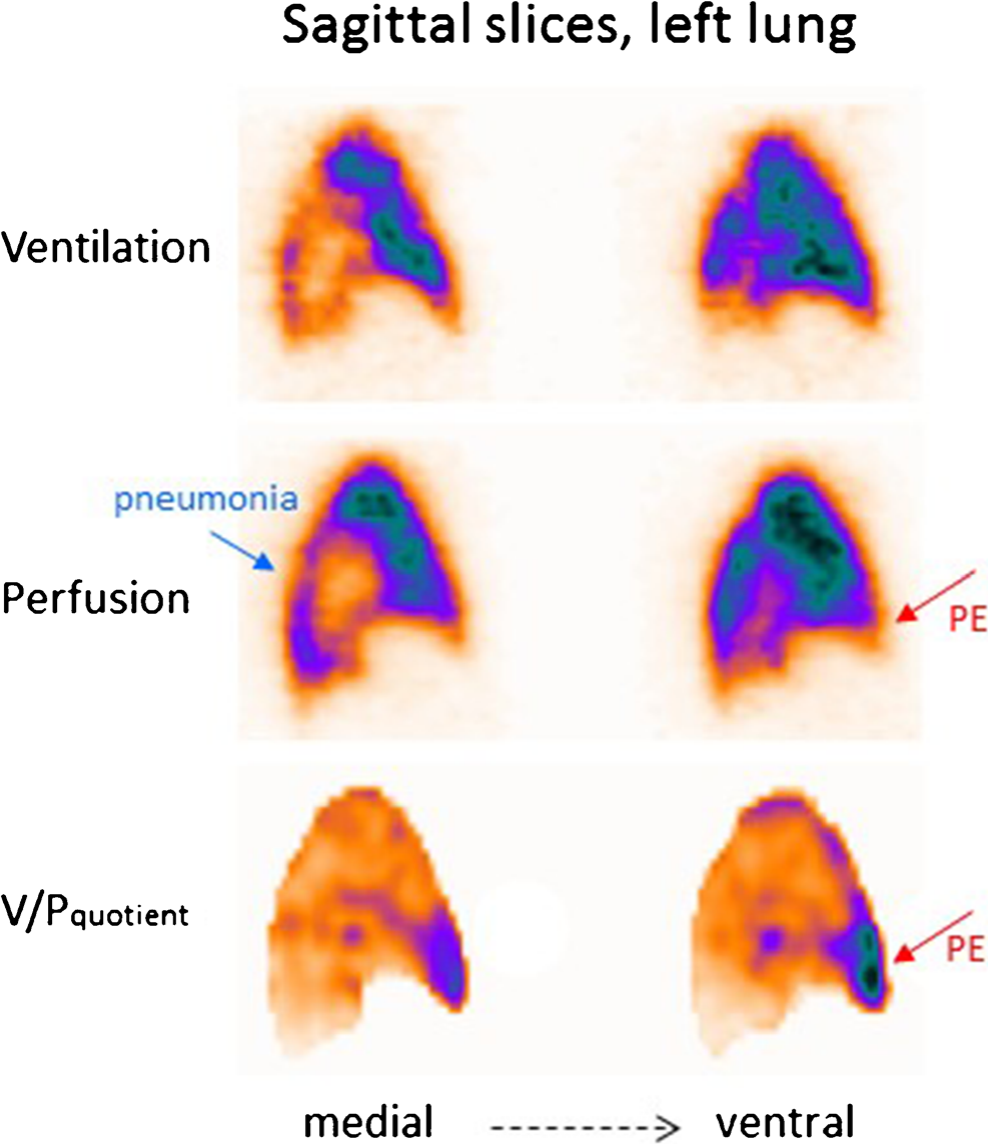
Sagittal slices of the left lung in a patient with PE in the medial lobe (red arrow) and pneumonia posteriorly (blue arrow). The VP mismatch may be highlighted in V/P quotient images

Some patients tend to develop recurrent episodes of PE. Figure 4 presents a patient with recurrent PE, shown by follow-up scans. Without both initial and follow-up images, it is often impossible to differentiate between old and new PE. The risks in patients treated with thrombolysis for massive PE include not only bleeding but also those related to unresolved PE. Immediate imaging control provides objective information on the need for repeated thrombolysis. Symptomatic patients with small emboli are diagnosed with sensitive methods, particularly V/P_SPECT_. Figure 5 presents a case with PE in the middle lobe and pneumonia in the posterior right lung which could not have been identified without SPECT and ventilation images.

The natural history and the efficacy of treatment in this group of patients is rather unknown. Thus, follow-up is indicated to individualise therapy [87–89].

The requirements that should be met by a method used for follow-up are as follows:

- Applicability to all patients
- Low radiation dose
- High sensitivity to allow estimation of resolution of even small emboli and occurrence of new ones

V/P_SPECT_ seems ideally suited for follow-up of PE because small and large emboli are both recognised, allowing a detailed study of regression or progression of the thrombotic disease [87–89]. Furthermore, the low radiation exposure enables repeat studies. Obviously, using the same method for diagnosis and follow-up is advantageous. Research in this area is especially indicated and a predischarge V/P_SPECT_ can be recommended to help identify patients in need of life-long treatment.

### V/P_SPECT/CT_

The introduction of integrated multimodality SPECT/CT cameras has enabled simultaneous acquisition of V/P_SPECT_ and CT, V/P_SPECT/CT_ [52–55, 90].

The CT is usually performed as a low-dose CT scan without contrast enhancement. The additional radiation dose is approximately 1–2 mSv, so that the whole V/P_SPECT/CT_ acquisition results in approximately 3–4 mSv. To reduce misalignment between the SPECT and CT images, it is proposed that CT scans are acquired during continuous shallow breathing [55]. Obviously, this will affect evaluation of structures particularly in basal regions, causing artefacts.

**Fig. 6:**
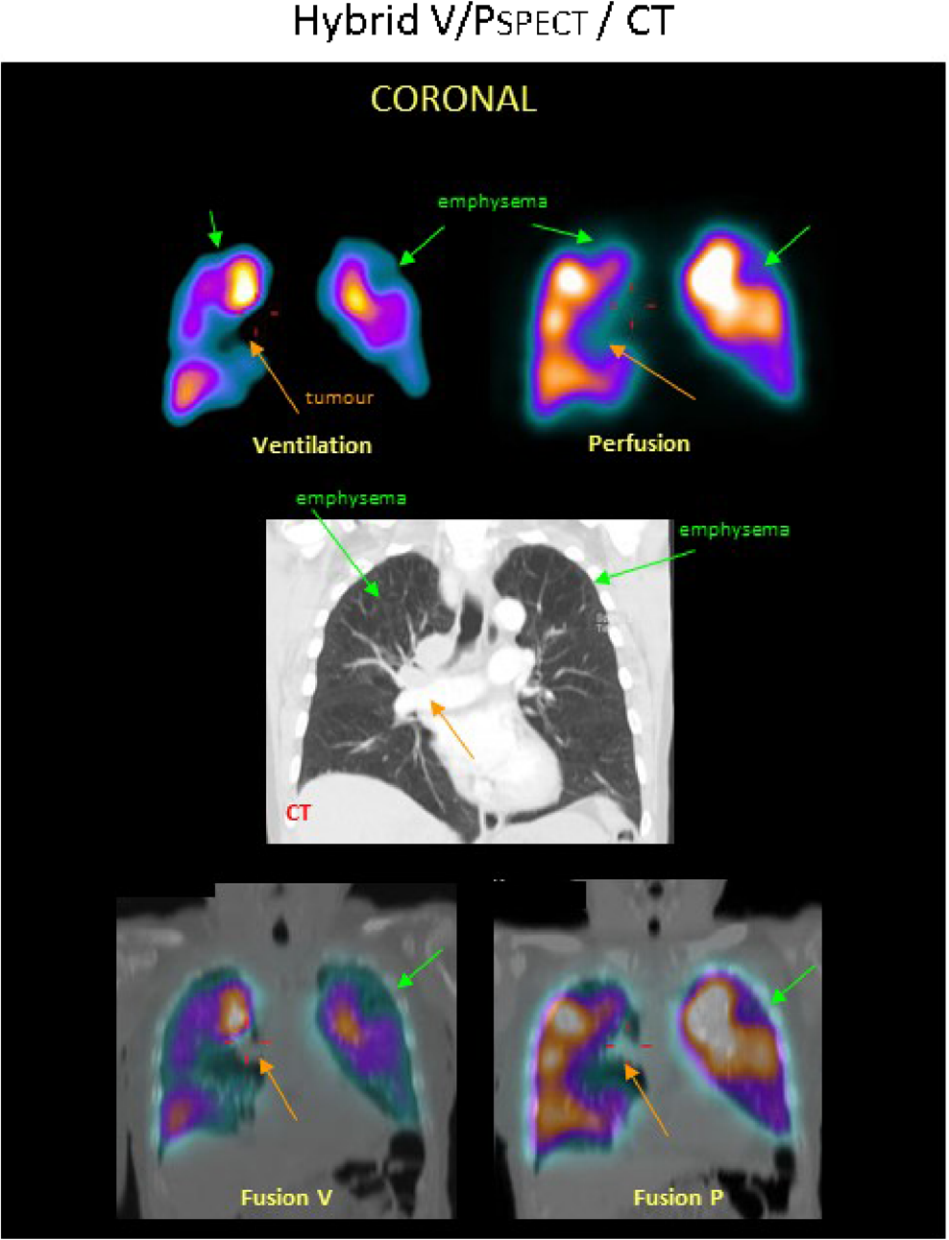
A patient with COPD, emphysema and tumour. Coronal slices display uneven distribution of ventilation with a pattern of deposition of ^99m^Tc-Technegas® that is typical for COPD. Perfusion follows the ventilation pattern. Matched ventilation and perfusion defects are observed in both upper lobes (green arrows) and to the right of the mediastinum (orange arrows). In the medial row of the corresponding coronal CT slice, emphysema is seen in both upper lobes (green arrows), as is a tumour in the mediastinum (orange arrow). Fusion images of CT and ventilation SPECT and CT and perfusion SPECT are shown

V/P_SPECT/CT_ has similar sensitivity as V/P_SPECT_ but slightly higher specificity for PE [52, 91]. Low-dose CT may visualise nonthromboembolic abnormalities such as emphysema, pneumonia and other parenchymal changes or extrinsic vascular compression, which may explain perfusion defects. Figure 6 presents a case with COPD, emphysema and a mediastinal tumour. CT acquired during a full-dose breathhold scan also has disadvantages with artefacts originating from the movement of the bronchi. CT image acquisition type is dependent on the capabilities of the scanner used.

Using low-dose CT instead of ventilation images approximates the sensitivity for PE compared with V/P_SPECT/CT_ but has a higher rate of false positives [52, 91, 92].

Further studies of V/P_SPECT/CT_ to define its value according to good clinical practice in various categories of patients seem merited. In patients with COPD, the added value of V/P_SPECT/CT_ has been validated [93].

### CTPA

Computed tomography angiography of the pulmonary artery (CTPA) is imaging of the pulmonary arteries during the passage of intravenously injected iodinated contrast material. Pulmonary emboli are visualsed as so-called filling defects caused by emboli within otherwise homogenously contrast-filled pulmonary arteries. CTPA is easy to perform in a few minutes.

CTPA needs to be embedded in decision strategies that are based on the assessment of clinical PE likelihood [31]. CTPA confirms the diagnosis of PE in clinically high probability patients (PPV > 95%). In the case of high pretest probability of PE and a negative CTPA, current data on diagnostic accuracy are inconsistent [94–96]; CTPA is overused in a great number of patients with low prevalence of PE [97, 98]. A PE located centrally in the pulmonary circulation can be detected by CTPA with a high PPV. The PPV decreases at segmental and subsegmental levels [96]. When clinical probability of PE is low or intermediate, CTPA may overdiagnose PE, leading to a low NPV [96].

CTPA has the potential to visualise additional pathologies other than PE such as pneumothorax, pneumonia, interstitial lung disease, pleural disease, aortic dissection and pathologies of the spine and rib cage. In the case of PE, CT is able to depict signs of right heart strain which is of prognostic importance [99–101].

### Risks of CTPA

The contraindications for CTPA are linked to the use of iodinated contrast media:

- *Iodine hypersensitivity*. Severe pseudoallergic reactions are generally very rare (approx. 0.04%, rarely fatal [102]). In an emergency scenario when CT cannot be withheld, intravenous premedication may be suitable.
- *Thyroid dysfunction*. Induction of hyperthyroidism and in rare cases also hypothyroidism is another complication associated with iodinated contrast media [103]. In an iodine-deficient geographical region, 2% of patients developed subclinical hyperthyroidism [104]. In unselected patients, overt hyperthyroidism occurred in 0.25% within 12 weeks [105]. Iodine-induced thyrotoxicosis is often overlooked in the elderly [106], and in the worst case, it may even cause a thyroid storm with cardiac arrest [107]. Risk factors are (potentially undiagnosed) Graves’ disease and/or thyroid autonomy [108]. Patients with thyroid storm should not receive iodinated contrast media at all; in other cases, premedication with oral sodium perchlorate (and thiamazole) may be helpful. Under emergency conditions, this treatment may be initiated directly after contrast exposure. Thyroid function is routinely monitored prior to contrast application in the vast majority of radiological institutions through serum baseline TSH.
- *Renal dysfunction*. The most severe complication of CTPA is contrast media-induced nephropathy (CIN) or contrast-induced acute kidney injury (CI-AKI). CI-AKI is defined as an increase in serum creatinine within 48–72 h after intravenous administration of low- or isoosmolarity iodinated contrast media. It carries a risk of chronic renal insufficiency, dialysis and death [100, 109]. The assumption of causality between intravenous contrast media administration and AKI has been challenged in recent publications, but data are controversial. The baseline glomerular filtration rate (eGFR) was found to be an independent predictor of AKI [110]. In two multivariate analyses, patients with an eGFR ≤ 43.6 mL/min [111] or eGFR < 60 mL/min [112], respectively, had the potential to develop CI-AKI. The odds ratios in the incidence of CI-AKI between contrast-enhanced CT and noncontrast CT increased below an eGFR < 30 mL/min [113]. In two meta-analyses, contrast-enhanced CT, compared with non-contrast-enhanced CT, was not significantly associated with AKI [114, 115]. In a large single-centre retrospective cohort study, the probability of developing AKI was 10.6%, 10.2% and 10.9% in the contrast-enhanced, unenhanced and non-CT group [116]. However, a selection bias must be considered as only a minority of patients with a baseline serum creatinine > 1.5 mg/dL received contrast media and factors that may have influenced the clinical decision to administer contrast media could not be perceived. Randomisation of patients to receive intravenous contrast media, once not considered ethically feasible, will be necessary to fully understand the role of contrast media in precipitation of renal dysfunction [116]. Current guidelines consider intravenous contrast administration to be safe to a creatinine clearance of 30 mL/min/1.73 m^2^ [117]. In almost every radiological institution, a baseline serum creatinine level and/or eGFR is determined prior to intravenous contrast administration to identify patients at risk. CI-AKI was considered to be the third most common cause in hospital acquired AKI [118]; now strong evidence is provided that the incidence of CI-AKI is substantially lower [109]. AKI is a multifactorial entity, and usually more than one risk factor (contrast media, nephrotoxic drugs, hypertension, age > 70 years, reduced cardiac output, diabetes mellitus and others) is involved [102].

### Comparison of V/P_SPECT_ and CTPA (see also Table 4)

#### Availability

CTPA is available in nearly all medical centres and community hospitals, often on a round-the-clock basis. V/P_SPECT_ is available in fewer hospitals and seldom on a 24-h basis. CTPA has been advocated for patients with suspected PE and signs of shock or hypotension [31].

**Table 4:** Summary of the pros and cons for the two principal methods to diagnose PE

	V/P_SPECT_	CTPA	Comment
Availability	Limited	Wide	Makes CTPA indispensable
Feasibility	Near 100%	Up to 50%	Makes V/P_SPECT_ indispensable
Rate of nondiagnostic studies	1–4% [68, 69, 119, 120]	4–10% [47, 96]	
Sensitivity	≥ 96%	≥ 78%	In high clinical probability, 40% needs further exam
Specificity	≥ 97%	≥ 98%	V/P_SPECT_ and CTPA equivalent
Effective radiation dose	1.2–2 mSv	4–20 mSv [121–127]	
Absorbed breast radiation dose	≈ 0.8 mGy	≈ 12–44 mGy [79, 128]	V/P_SPECT_ crucial for young women
Additional diagnoses	Common, important	Common, important	Highest documented rate—V/P_SPECT_
Diagnostics of chronic pulmonary embolism	Reference method?	Not useful but needed prior to surgery?	V/P_SPECT_ is gold standard
Follow-up and research	Optimal		V/P_SPECT_ offers quantitative data

#### Feasibility and rate of nondiagnostic studies

Feasibility of CTPA is restricted by the need to use intravenous iodinated contrast media. Whilst the general risk of CI-AKI seems to be overestimated [109] and iodine-induced thyrotoxicosis may be prevented by premedication, the residual risks are of a serious nature. Elevated serum creatinine and a suppressed TSH are relative contraindications to CTPA provided that V/P_SPECT_ is available [52, 96]. High flow rates of contrast medium are a precondition for CTPA, but cannot be achieved in all patients. Motion artefacts reduce spatial resolution. In the PIOPED II trial, CTPA was inconclusive in 6.2% of patients because of poor image quality, in a subgroup this was 10.6% [96]. An increase of detector rows in CT improves resolution of CTPA, but the number of inconclusive results remained at 10% in both the 4-row and 64-row cohort, mostly due to movement artefacts and suboptimal contrast opacification [47]. In pregnant patients and those with a very low prevalence of PE (3.3%), inconclusive results were seen in 5.9% [129]. Nondiagnostic studies with V/P_SPECT_ may be obtained with conventional radioaerosol in patients with severe COPD, but this restriction has been mostly eliminated by the use of Technegas® [68, 69, 119, 120]. In conclusion, if contrast media-related risks are considered, feasibility of CTPA is restricted in many more patients than with V/P_SPECT_.

In haemodynamically unstable patients, CTPA is often recommended [31, 130]. However, if a gamma camera is available, perfusion-only scintigraphy, even a single planar image, is adequate to exclude massive PE [130].

#### Accuracy of PE diagnosis

The lack of a satisfactory gold standard for making the diagnosis of PE poses difficulties for the assessment of sensitivity, specificity and accuracy of all diagnostic methods. Follow-up of patients for recurrence of PE as a predictor of false negative results may overcome this limitation. The comparison of V/P_SPECT_ and CTPA shows controversies. On one side, no performance difference between V/P_SPECT_ and CTPA was seen [121]. In other studies, V/P_SPECT_ is proposed to be superior to CTPA in cases with other underlying lung diseases preventing the diagnosis of PE with CTPA. V/P_SPECT_ more often provided a diagnosis of PE in patients with high clinical suspicion of PE and in the presence of indeterminate CTPA imaging (sensitivity 93% vs. 83%) [68, 94, 131, 132].

Another available benchmark is follow-up of patients for recurrence of PE as a predictor of false negative results. An equivalent of sensitivity was calculated on the basis of a 3-month follow-up in 14,545 patients where PE was excluded by CTPA [48, 94, 95, 133–138] and in another 1865 patients where this was done by V/P_SPECT_ [52, 74, 75, 139]:

$$ \mathrm{Sensitivity}\ \left(\mathrm{equivalent}\right)\left[\%\right]=\frac{\mathrm{PE}\ \mathrm{prevalence}\ \left[\%\right]-\mathrm{VTE}\ \mathrm{relapse}\ \mathrm{rate}\ \left[\%\right]}{\mathrm{PE}\ \mathrm{prevalence}\ \left[\%\right]}\bullet 100 $$

The mean sensitivity (equivalent) was > 95% for the both methods. The results do not reflect true sensitivity of the imaging test, since most PE are single events, not recorded by follow-up. On the other hand, sensitivities derived from relapse rates clearly indicate that both V/P_SPECT_ and 16- to 64-row CTPA are able to recognise larger PE with a tendency to recurrent episodes of PE. An error rate of about 5% may be caused by false negative results of the imaging test with subsequent PE relapse, but may also reflect true negative results with subsequent primary PE events. Large multicentre management outcome studies based on V/P_SPECT_ with a standardised diagnostic algorithm defined a priori are needed to differentiate this.

In a systematic review and meta-analysis, diagnostic performance of V/P_SPECT_ was equivalent to CTPA [121]. In head-to-head comparison studies, V/P_SPECT_ was superior to CTPA using ROC analysis [140]. This applies particularly in cases with other underlying lung diseases and when the diagnosis of PE could not be established with CTPA [68, 94, 132, 141]. V/P_SPECT_ more often provided a diagnosis of PE in patients with a high clinical suspicion of PE and in the presence of indeterminate CTPA (sensitivity 93% vs. 83%) [131]. Superior sensitivity of V/P_SPECT_ is best seen in patients with chronic pulmonary embolism where V/P_SPECT_ has reference status [142], but CTPA and right heart catheterisation are also essential for patient care.

In conclusion, both techniques display specific advantages and shortcomings. V/P_SPECT_ is superior to CTPA in cases with underlying other lung diseases and when the diagnosis of PE cannot be established with CTPA [68, 94, 131, 132].

### Radiation exposure

A key objective of imaging PE is to minimise radiation exposure without sacrificing image quality and diagnostic accuracy. The amounts of radiation involved must be considered together with imaging protocols. Table 1 gives basic data on radiation exposure for V/P_SPECT_. With the recommended activities for ventilation and perfusion, the effective dose is 2 mSv.

In a systematic review and meta-analysis of the literature, radiation exposure was 2.12 mSv for V/P_SPECT_ per correct diagnosis compared with 4.96 mSv for CTPA [121]. In clinical routine, radiation doses between 3.5 and 13.2 mSv have been reported for CTPA [122–124]. Automated data collection as volume CT dose index and dose-length product, averaged to effective doses, varies considerably within and across facilities. Primary factors that influence dose variations are patient size (weight and chest diameter), multiphase scanning and institutional protocol choices [125]. In general, higher effective doses (> 5 mSv) are reported from automatically collected data [125–127].

The most critical organ in CTPA is the female breast. Absorbed radiation doses to the female breast ranging from 8.6 to 44 mSv have been reported [79, 128, 143]. Tube current modulation is able to decrease the breast dose from 51.5 to 8.6 mSv [143], whilst shielding is less effective [144]. Absorbed radiation dose to the female breast from V/P_SPECT_ is < 1 mSv [79]. Fetal-absorbed doses for V/P_SPECT_ and CTPA are similar and so small that they are unlikely to be clinically significant [79, 128, 145].

In conclusion, effective and absorbed doses are lower for V/P_SPECT_ than for CTPA. The difference is of particular importance for the female breast, particularly in young women and critically so during pregnancy. Fetal doses are low and similar for both methods.

## Additional diagnostic contributions of V/P_SPECT_

### Chronic obstructive pulmonary disease

**Fig. 7:**
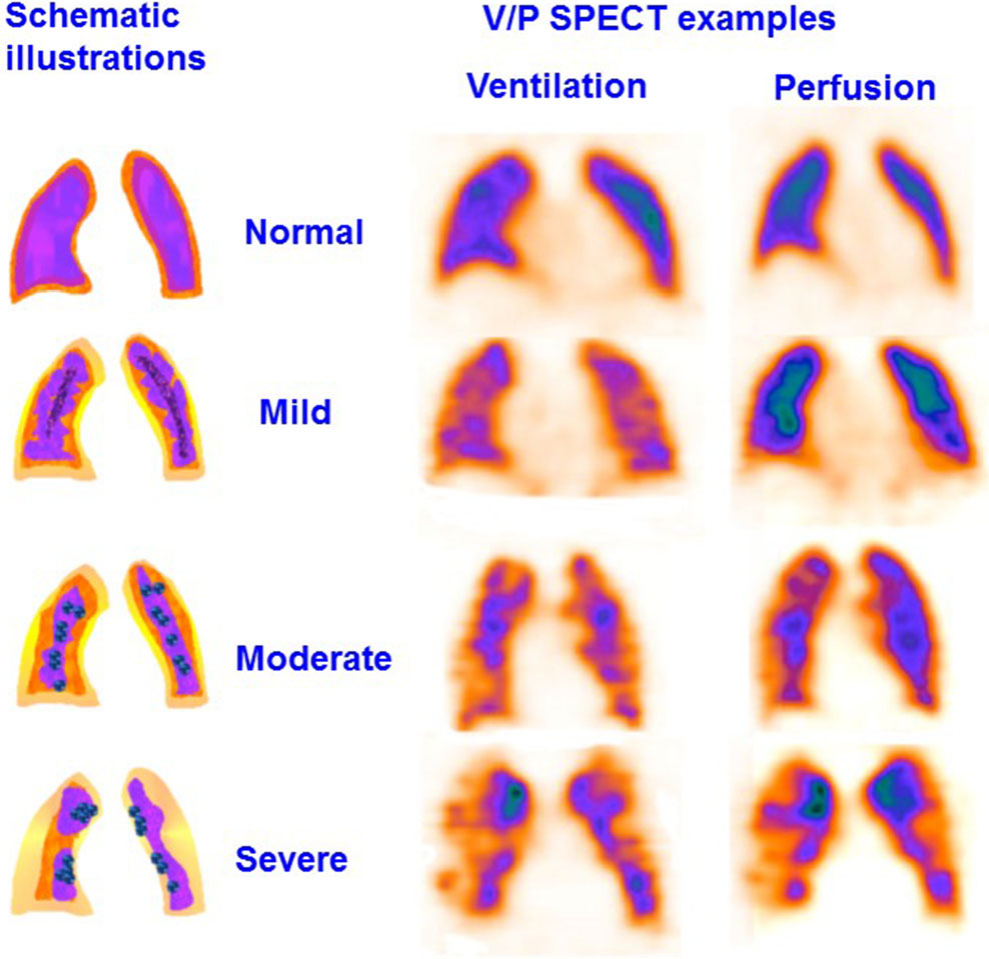
Schematic presentation of the obstructive lung disease grading system and correlating representative V/P_SPECT_ images that shows different degrees of airway obstruction on coronal slices. 0: normal, even distribution of Technegas® with good peripheral penetration and without accumulation in large or small airways. 1: mild airway obstruction, slightly uneven distribution with some deposition of aerosol in small and intermediate airways. Only minor areas with reduced peripheral penetration are observed. 2: moderate airway obstruction, deposition of Technegas® in intermediate and large airways, diminished peripheral penetration with maximum accumulation in the central half of the lung. 3: severe airway obstruction, central deposition in large airways with severely impaired penetration of Technegas® and major areas with reduced or abolished function

**Fig. 8:**
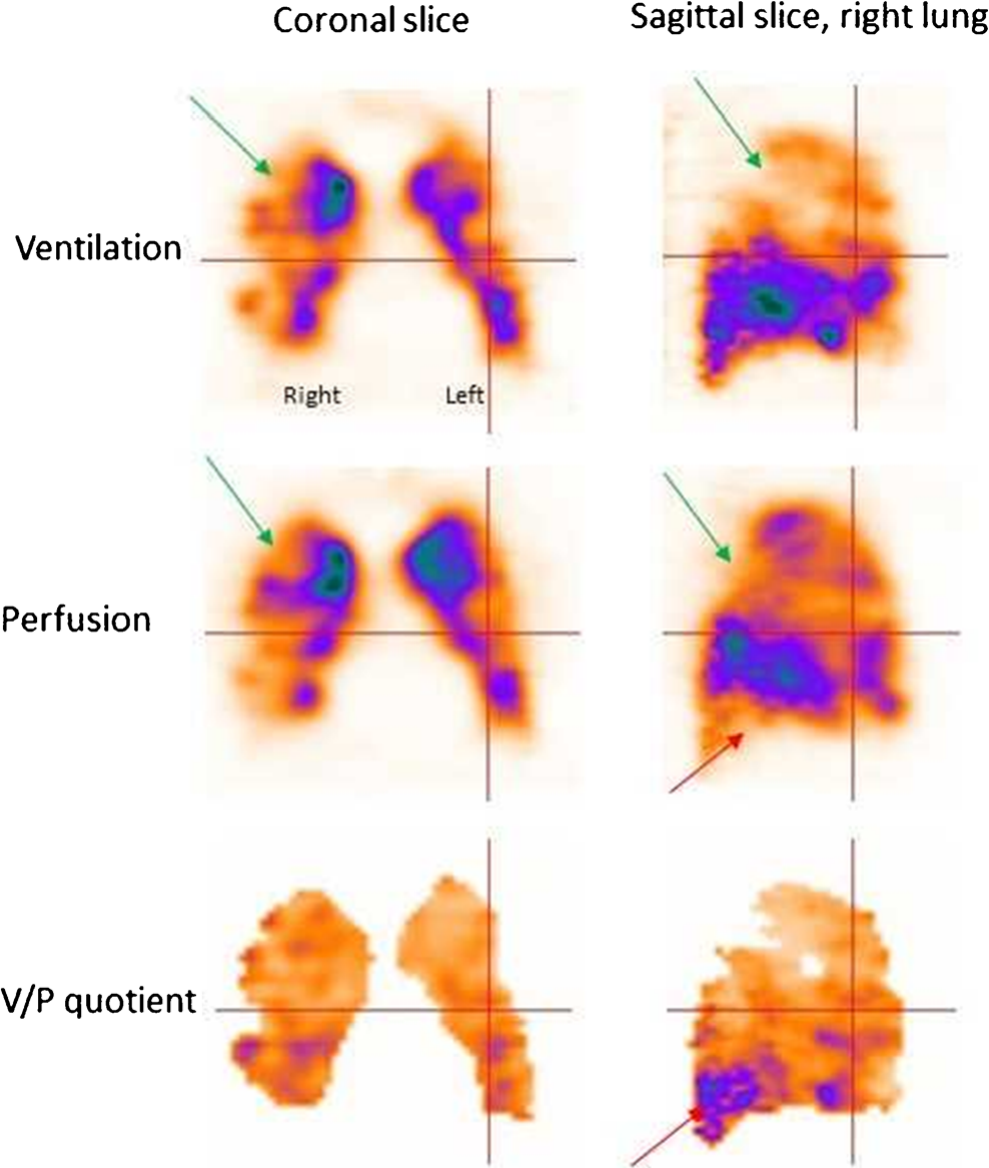
V/P_SPECT_ images showing a severe degree of airway obstruction in coronal and sagittal projections in a patient with severe COPD, emphysema (green arrow) and PE (red arrow)

A general unevenness of ventilation typical for COPD has been observed in V/P_SPECT_ in patients investigated for PE [68, 120, 146]. Perfusion is usually less affected, which leads to so-called reversed mismatch [62, 93, 119]. The degree of ventilation defects reflects varying degrees of obstruction with COPD. Figure 7 presents cases with different degrees of COPD [62, 68, 120, 147–149]:

- Grade 1: uneven aerosol distribution through the lung seen in mild COPD.
- Grade 2: uneven aerosol distribution and reduced Technegas® penetration to the periphery, with deposition of aerosols in small airways, seen as hotspots. This indicates moderate COPD.
- Grade 3: a severely impaired Technegas® penetration to the periphery and a central deposition of Technegas® in large airways, usually with large areas of reduced/absent ventilation. This indicates severe COPD. Figure 8 presents a case with severe COPD, emphysema and PE.

### Left heart failure

**Fig. 9:**
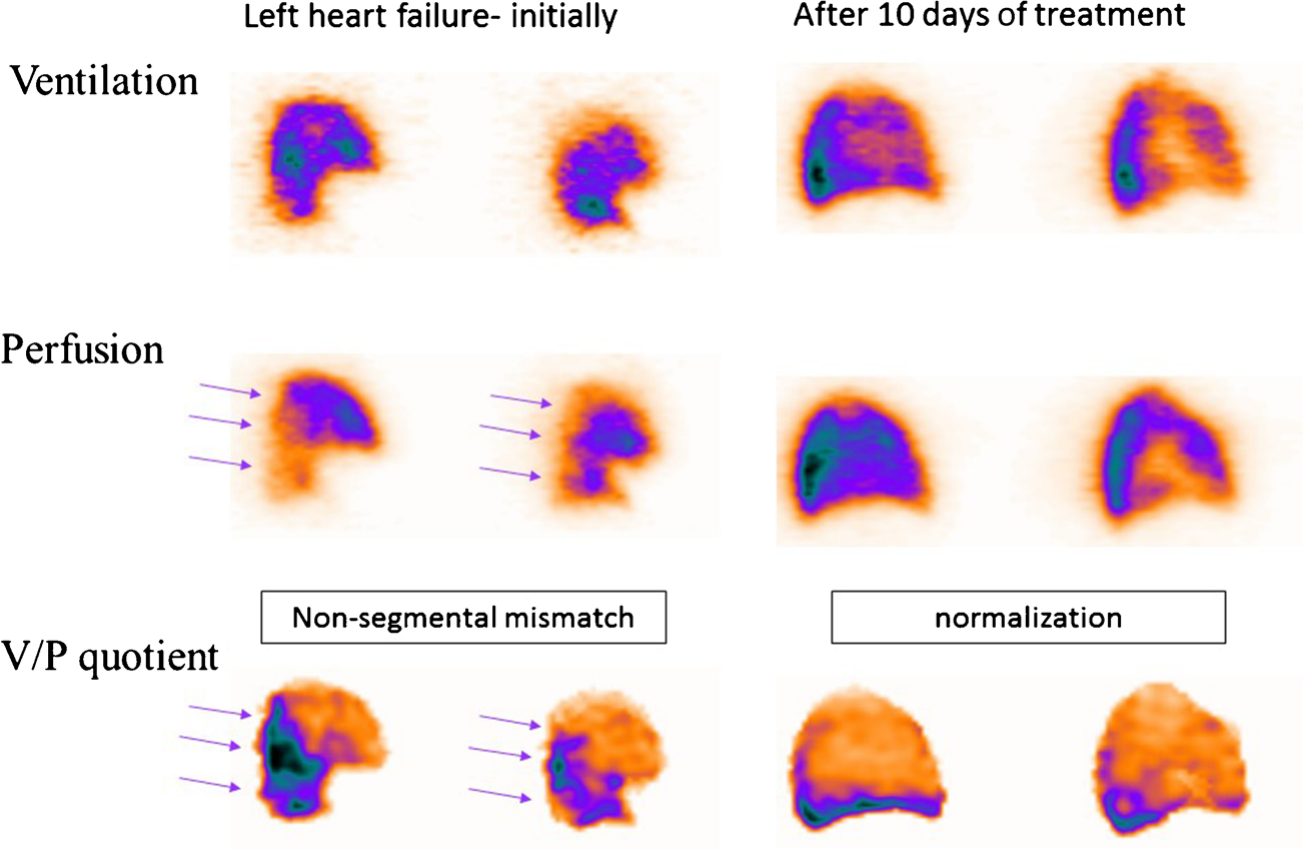
Sagittal slices from the lung show antigravitational redistribution of perfusion in left heart failure. Ventilation is less affected causing mismatch. Mind the pattern; it is not of segmental character!

Antigravitational perfusion distribution is typical for pulmonary congestion due to left heart failure. In supine patients, redistribution of perfusion towards anterior regions has been observed in 5–15% of patients with suspected PE [146, 150–152]. Based upon a measured vertical perfusion gradient, a positive predictive value of ≥ 88% to detect pulmonary congestion has been reported [151]. As ventilation is usually less affected, V/P mismatch may be observed in dorsal regions. Figure 9 presents a case with left heart failure initially and at the follow-up. This mismatch has a nonsegmental pattern (Fig. 9). It does not conform to the anatomy of pulmonary vascular architecture and should, therefore, not be misinterpreted as PE. V/P_SPECT_ was recently validated against right heart catheterisation for diagnosis of pulmonary congestion in left heart failure [153].

### Pneumonia

**Fig. 10:**
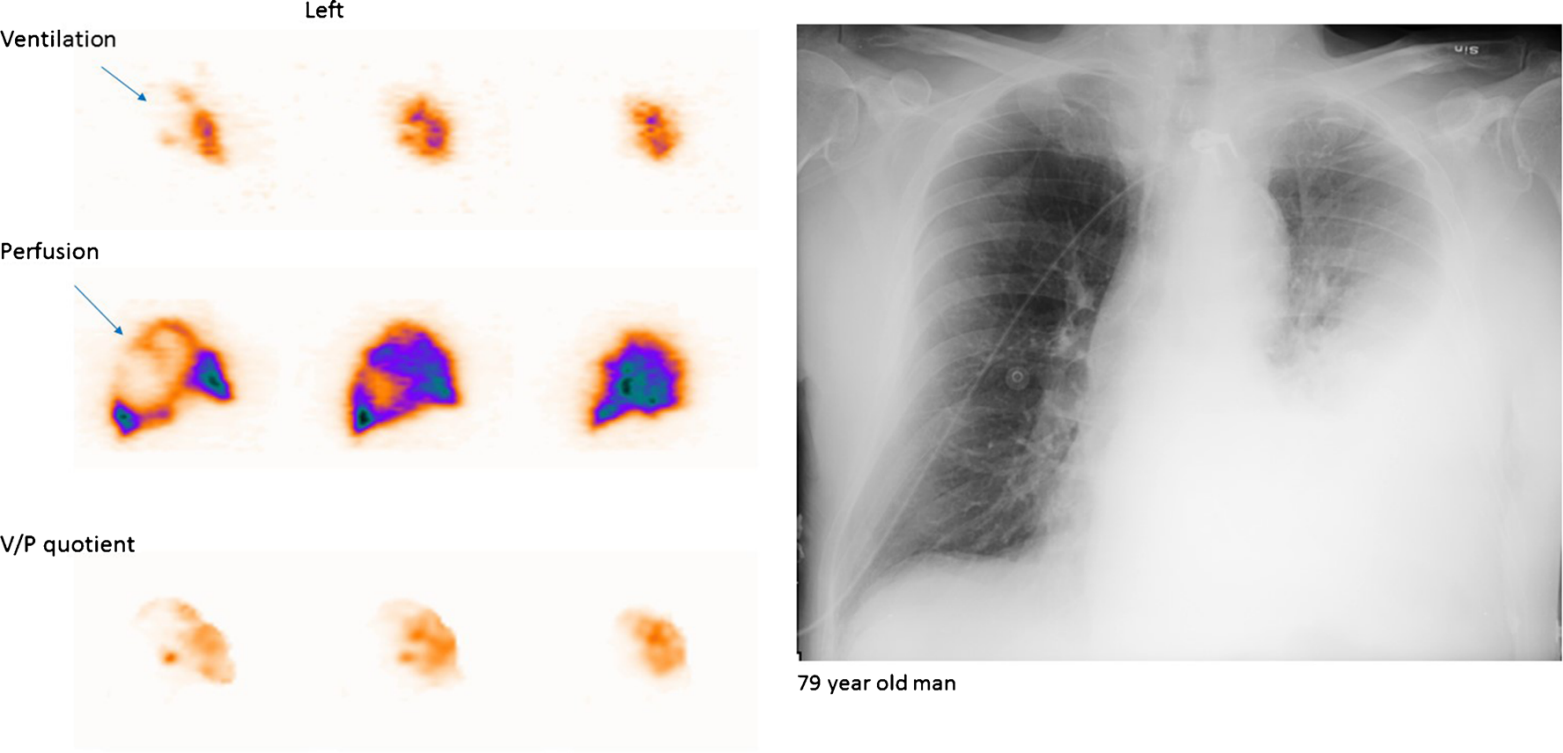
Sagittal slices of the left lung in a patient with extensive pneumonia in whom the chest X-ray was interpreted as showing atelectasis (**a**). The left lung shows nearly absent ventilation in areas with much better perfusion. Arrow indicates stripe sign. V/P quotient highlight reverse mismatch

Pneumonia is a general term for conditions of lung inflammation often caused by bacterial, viral or fungal infections. It presents with nonspecific symptoms, and like other illnesses, this can lead to diagnostic problems [154]. V/P_SPECT_ shows ventilation defects, which usually exceed perfusion defects (reversed V/P mismatch) [155, 156]. Preserved perfusion along the pleural border recognised as the ‘stripe sign’ is a specific sign of pneumonia [1, 157, 158]. Figure 10 presents a case with pneumonia on V/P_SPECT_ and chest X-ray. Such ventilation-perfusion patterns in pneumonia have been documented with positron emission tomography as well [159]. Reversed-mismatched or matched V/P defects typical for pneumonia can be found in V/P_SPECT_ in patients who are studied for PE. Figure 5 presents a case with PE and pneumonia. In some patients, V/P defects typical for pneumonia reduce the total lung function in the absence of any structural CT defects [146, 160]. Nevertheless, the potential of V/P_SPECT_ for diagnosis and management of pneumonia has in general not been exploited.

### Chronic pulmonary embolism and chronic thromboembolic pulmonary hypertension

**Fig. 11:**
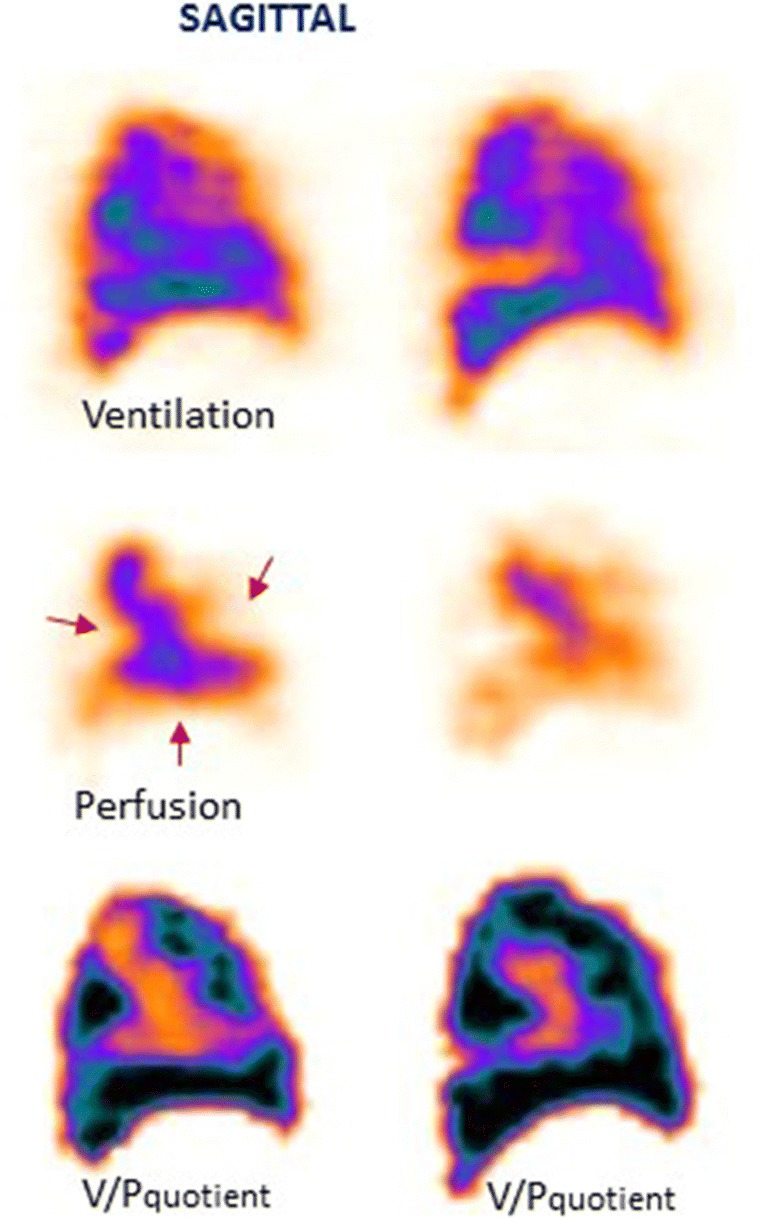
Patient with chronic PE. The perfusion is only maintained in the central part of the lung, leading to large nonsegmental perfusion defects (red arrows)

Chronic PE represents a condition in which perfusion defects due to pulmonary emboli have not resolved. Its clinical presentation is often insidious. It might be progressive and can lead to chronic thromboembolic pulmonary hypertension (CTEPH), right heart failure, arrhythmia and death. Figure 4 presents a case with recurrent PE and CTEPH. Figure 11 presents a case with chronic PE. CTEPH might be a consequence of repeated unrecognised small PEs. The incidence of CTEPH secondary to acute PE is around 5% [27, 142, 161–163].

V/P_scan_ with a sensitivity of 96% and specificity of 90% is a mainstay in the diagnosis of CTEPH, since CTPA has a sensitivity of only ≈ 50% [164]. The higher sensitivity of V/P_SPECT_ compared with CTPA has been confirmed [165]; agreement between CTPA and scintigraphy ranged from fair (*κ* = 0.31) to slight (*κ* = 0.09) [166].

V/P_scan_ is the imaging test of choice to exclude CTEPH [142], but is underused to diagnose CTEPH [167–169]. Pulmonary scintigraphy might differentiate among different types of CTEPH (Fig. 6).

After diagnosis of CTEPH, CTPA and right heart catheterisation are important for management decisions about therapy.

### Lung cancer radiotherapy planning

Radiation therapy, alone or in combination with other treatment, plays an important role in the management of lung cancer. ‘Functional image-guided lung avoidance radiotherapy’ is an emerging concept aimed at delivering a high radiation dose to the lung cancer tumour volume whilst minimising irradiation to the uninvolved functional lung tissue. In the future, V/P_SPECT_ could become an interesting technique to provide the functional information needed for the planning of such treatment [170].

## Pregnancy

Pregnancy poses unique circumstances in diagnosing PE:

- The incidence of PE in pregnancy is about fivefold higher than in nonpregnant females of a similar age and is the leading nonobstetric cause of death during pregnancy in developed countries. The incidence of PE and DVT is about 1 and 3‰, respectively [171]. The incidence is similar in all 3 trimesters [172]. The diagnostic accuracy of any test is compromised by a low prevalence of PE in this collective [147, 173].
- d-dimer is not useful because it is elevated during pregnancy [174]. CTPA has a high rate of nondiagnostic tests due to changed haemodynamics [175–179].
- CTPA leads to unique radiation hazards to the maternal breast [180].

### Imaging tests

#### Ultrasonography

To avoid unnecessary irradiation, venous compression ultrasonography can be considered. However, the diagnostic yield can be low [145].

#### CTPA

An increased blood volume and cardiac output shortens the arrival time of intravenous contrast in the pulmonary vessels, necessitating adjustments in triggered scan delays [178, 181]. Transient influx of unopacified blood from the inferior vena cava has also been identified as a cause for poor-quality CTPA [178, 182]. Nondiagnostic CTPA scans occur in 6–36% of patients, whilst alternative diagnoses were identified in 2–13% [145, 178, 183, 184]. Prenatal exposure to iodinated contrast media is not a risk factor for neonatal thyroid dysfunction [185].

#### V/P_SPECT_

To minimise radiation, a 2-day protocol is recommended. Perfusion-only SPECT is performed on day 1, with only 50 MBq ^99m^Tc-MAA. Because of the low incidence of pulmonary disorders in pregnant women, PE is usually excluded based on a normal perfusion pattern [186]. In the case of an abnormal perfusion pattern, anticoagulation therapy can be started until a ventilation study is performed on day 2, using a lung-deposited activity of 20–30 MBq. This strategy leads to a high sensitivity and specificity of the examination [145, 147]. After the first trimester, the standard 1-day V/P_SPECT_ protocol may be considered.

### Dosimetry

Fetal doses for both CTPA and perfusion SPECT are ≤ 0.12 mGy [145, 187–190]. The maternal absorbed dose, mainly to the breast, differs significantly and may range from 5 to 20 mSv for CTPA [121, 125, 127, 145, 191, 192] and 0.5–0.8 mSv for V/P_SPECT_ [145, 147].

### Recommendation

If both imaging modalities are available, V/P_SPECT_ is recommended due to the following:

- Near 100% accuracy of the diagnostic test
- The considerably lower breast radiation dose [79, 128, 147, 189, 193]
- No contraindications

### Diagnostic algorithm

The likelihood of PE is assessed using a clinical prediction tool and, where indicated, the measurement of d-dimer. Where acute PTE is suspected, patients should be treated with heparin (unless contraindicated) until the test result is known. The choice of imaging test will depend on local availability but where possible V/P_SPECT_ is preferred over VP_PLANAR_. Few cases using V/P_SPECT_ are nondiagnostic, and in such instances, a further test (CTPA) is necessary to confirm or exclude PE. V/P_PLANAR_ has a higher indeterminate rate, in which case further investigation (usually CTPA) is necessary for diagnosis. At negative CTPA, further test should be performed in patients with remaining clinical suspicion of PE (blue arrow). With regard to the availability of V/P (planar or SPECT), it is highly variable depending on the hospital and country, but in many centres, the availability is not 24 h a day nor every day of the week/year.
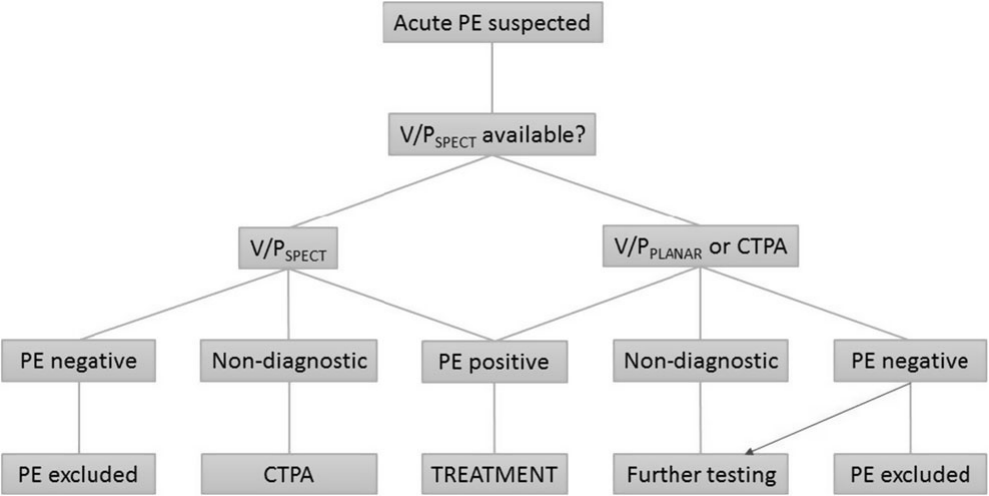

1. ‘This guideline summarizes the views of the Cardiology Committee of the EANM/European Association of Vascular Medicine, experts in radiology and cardiology, and reflects recommendations for which the EANM cannot be held responsible. The recommendations should be taken into context of good practice of nuclear medicine and do not substitute for national and international legal or regulatory provisions’.
2. ‘The guidelines were brought to the attention of all other EANM Committees and to the European National Societies of Nuclear Medicine. The comments and suggestions from the EANM Committees and from the European National Societies are highly appreciated and have been considered for this Guideline’.

## Abbreviations

AKI: Acute kidney injury
COPD: Chronic obstructive pulmonary disease
CTPA: Computed tomography pulmonary angiography
CIN: Contrast media-induced nephropathy
CTEPH: Chronic thromboembolic pulmonary hypertension
DTPA: Diethylene triamine pentaacetic acid
DVT: Deep venous thrombosis
EANM: European Association of Nuclear Medicine
ELISA: Enzyme-linked immunosorbent assay
ESC: European Society of Cardiology
^81m^Kr: Radioactive krypton gas
MAA: Macroaggregated human albumin
NPV: Negative prediction value
OSEM: Ordered subset expectation maximisation
PE: Pulmonary embolism
PERC: Pulmonary embolism rule-out criteria
PIOPED: Prospective investigation of pulmonary embolism diagnosis
PPV: Positive predicitive value
SNMMI: The Society of Nuclear Medicine and Molecular Imaging
^99m^Tc-DTPA: ^99m^Tc-diethylen-tetraamino-pentaacetate
V/P_PLANAR_: Ventilation and perfusion scan with planar imaging
V/P_SCAN_: Ventilation and perfusion scan
V/P_SPECT_: Ventilation and perfusion single-photon emission tomography
VTE: Venous thromboembolism
